# Restoration of upper limb movement via artificial corticospinal and musculospinal connections in a monkey with spinal cord injury

**DOI:** 10.3389/fncir.2013.00057

**Published:** 2013-04-11

**Authors:** Yukio Nishimura, Steve I. Perlmutter, Eberhard E. Fetz

**Affiliations:** ^1^Department of Physiology & Biophysics, University of WashingtonSeattle, WA, USA; ^2^Washington National Primate Research Center, University of WashingtonSeattle, WA, USA; ^3^Precursory Research for Embryonic Science and Technology, Japan Science and Technology AgencyTokyo, Japan

**Keywords:** brain–computer interface, artificial neural connection, hand, spinal cord injury, local field potential, muscle, spinal cord, monkey

## Abstract

Functional loss of limb control in individuals with spinal cord injury or stroke can be caused by interruption of corticospinal pathways, although the neural circuits located above and below the lesion remain functional. An artificial neural connection that bridges the lost pathway and connects cortical to spinal circuits has potential to ameliorate the functional loss. We investigated the effects of introducing novel artificial neural connections in a paretic monkey that had a unilateral spinal cord lesion at the C2 level. The first application bridged the impaired spinal lesion. This allowed the monkey to drive the spinal stimulation through volitionally controlled power of high-gamma activity in either the premotor or motor cortex, and thereby to acquire a force-matching target. The second application created an artificial recurrent connection from a paretic agonist muscle to a spinal site, allowing muscle-controlled spinal stimulation to boost on-going activity in the muscle. These results suggest that artificial neural connections can compensate for interrupted descending pathways and promote volitional control of upper limb movement after damage of descending pathways such as spinal cord injury or stroke.

## INTRODUCTION

Functional loss of limb control in individuals with spinal cord injury or stroke can involve interruption of descending pathways to spinal networks, although the neural circuits located above and below the lesion retain their function. An artificial neural connection that bridges the lost pathway has potential to compensate for the functional loss. Recent studies showed that monkeys could use cortical activity to control functional electrical stimulation (FES) in muscles transiently paralyzed by nerve block (Moritz et al., 2008; Pohlmeyer et al., 2009; Ethier et al., 2012). However, restoring coordinated movement of paralyzed limbs with peripheral FES remains problematic (Popovic et al., 2002). Stimulation of peripheral nerve or muscle often evokes movement about only a single joint and recruits the largest, most fatigable motor units first. Spinal microstimulation offers an alternative method to produce coordinated movement and more natural recruitment of motor units (Mushahwar and Horch, 2000; Mushahwar et al., 2000; Mussa-Ivaldi and Bizzi, 2000; Saigal et al., 2004; Moritz et al., 2007). In anesthetized animals, current can be delivered to spinal sites to produce coordinated patterns of muscle contraction (Zimmermann et al., 2011).

Several studies have demonstrated that multichannel spike signals recorded with intracortical electrode arrays can be used to estimate arm movements (Kennedy et al., 2000; Wessberg et al., 2000; Serruya et al., 2002; Carmena et al., 2003; Choi et al., 2009; Vargas-Irwin et al., 2010) and muscle activity (Morrow and Miller, 2003; Santucci et al., 2005; Koike et al., 2006; Pohlmeyer et al., 2007; Schieber and Rivlis, 2007). Although recordings of cell assemblies by intracortical electrodes can provide a rich repertoire of signals, their limitations include signal deterioration due to glial scarring (Polikov et al., 2005), potential displacement from the recording site (Leuthardt et al., 2004) and invasive recording techniques. Chronically implanted electrode arrays typically lose the ability to record cell spikes after several years (Krüger et al., 2010; Simeral et al., 2011). Reliable spike recording is a challenge for the long durations required for clinical applications. Movement parameters can also be decoded from local field potentials (LFPs; Zhuang et al., 2010; Flint et al., 2012) and the electrocorticogram (Schalk et al., 2007; Sanchez et al., 2008; Miller et al., 2009; Chao et al., 2010; Shin et al., 2012) in motor-related areas, potentially offering more stable signals that represent the activities of many neurons near the electrode. Thus, instead of relying on cell spikes recorded with intracortical electrodes, it is possible to use cortical field potentials, or muscle activity as a surrogate of cortical cell activity.

Here we describe a case study in which an awake monkey with spinal cord injury could volitionally control the paretic upper limb through artificial neural connections using LFPs in motor cortex or activity of muscles to trigger stimulation of a spinal site appropriate to restore goal-directed movement of the affected arm.

## MATERIALS AND METHODS

Experiments were performed with a male *Macaca nemestrina* monkey (4 years old, weight 5.5 kg). The experiments were approved by the Institutional Animal Care and Use Committee (IACUC) at the University of Washington and all procedures conformed to the National Institutes of Health Guide for the Care and Use of Laboratory Animals.

### SURGERIES

All implant surgeries were performed using sterile techniques while the animal was anesthetized using 1–1.5% sevoflurane. Dexamethasone, cephalexin, and ketoprofen were administered preoperatively and buprenorphine was given post-operatively.

#### Cortical implants

**FIGURE 1:**
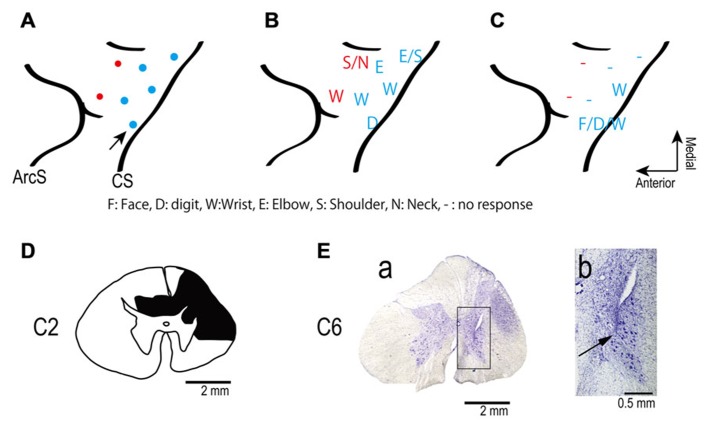
**(A)** Electrode locations in the motor areas of the lateral aspect of the frontal lobe of the left (contralesional) hemisphere. Electrodes were placed in primary motor cortex (blue dots) and in dorsal premotor cortex (red dots). **(B,C)** Somatotopic map shows movements evoked from each site in frontal lobe before **(B)** and after **(C)** spinal cord injury. The pre-lesion maps were established by ICMS at movement threshold (20–120 μA). The post-lesion maps were established by ICMS at 450 μA on post-lesional day 14. The maps show the region between the central sulcus (CS: diagonal line to the right of each panel) and the arcuate sulcus (ArcS: curved line to the left). Arrow indicates site used in session illustrated in **Figure 3**. **(D)** Drawing of the C2 segments showing the extent of the spinal cord lesion (hatched in black). **(E)** Electrode location in spinal cord. **(a)** Electrodes were targeted at the ventral horn and intermediate zone of the spinal cord. **(b)** Higher magnification view of the location of an electrode tip (black arrow).

Silver electrode wires (0.1 mm diameter, ~50 kømega at 1 kHz) were chronically implanted after making small incisions in the dura, in the digit, wrist and arm areas of primary motor cortex (M1) and the arm area of the dorsal aspect of premotor cortex (PMd) in the left hemisphere (contralateral to the spinal lesion, **Figure 1A**). The incisions of the dura mater were sutured closed. Small titanium-steel screws were attached to the skull as anchors. A stainless steel head-post was mounted on the skull for head fixation. The cortical electrodes and the head-post chamber were anchored to the screws with acrylic cement.

#### Surgery for EMG recording

Initially, electromyographic (EMG) activity was measured with electrodes surgically implanted in 16 arm and hand muscles, identified by anatomical features and by movements evoked by trains of low-intensity stimulation. Bipolar, multistranded stainless steel wires (Cooner Wire, Chatsworth, CA, USA) were sutured into each muscle and wires were routed subcutaneously to a connector on the animal’s back. A jacket worn by the monkey prevented access to the back connector between recording sessions. After these electrodes were broken by the monkey, additional wires were implanted transcutaneously for subsequent EMG recordings.

#### Surgery for spinal implant and spinal cord lesion

We made two separate unilateral laminectomies on the right side in the same surgery to prepare to record the activity of spinal neurons during behavior. The laminae and dorsal spinous processes of the C2–C3 and C5–C7 vertebrae were removed. A chamber was implanted over the rostral laminectomy. Stimulus electrodes were implanted at the caudal site. After recovery from surgery the monkey exhibited an upper arm hemiparesis, including inability to control the digits independently, and the recordings were not performed. The deficit remained throughout the 3 months during which these experiments were performed. Post-mortem histology revealed that a spinal cord lesion was inadvertently created around C2 to C3 during surgery, perhaps due to a contusion on the spinal surface or hemorrhage (**Figure 1D**).

For the stimulus electrode, the dura mater and arachnoid under C5–C7 vertebrae were removed. Eleven polyurethane-coated, platinum–iridium wires (diameter 30 μm; impedance 200–600 kømega at 1 kHz) were inserted 2.5–4 mm into the lower-cervical spinal cord targeting the ventral horn where hand motoneurons are located (Jenny and Inukai, 1983; Chiken et al., 2001). Penetration depth was determined by making a sharp bend in the microwire at the appropriate length. A second bend several mms more proximal provided strain relief and allowed the wires to float on the cord (Mushahwar et al., 2000). The microwires were bonded with cyanoacrylate glue to the spinal surface at each penetration point. The wires were routed into a silicone tube, which was glued with dental acrylic to bone screws placed in the lateral masses and T1 dorsal process, and routed through the skin to a connector. The spinal cord was covered with the subcutaneous fascia and gel-foam. The laminectomy was closed with acrylic cement. The skin and underlying soft tissue were then sutured closed.

### TORQUE-TRACKING TASK

Prior to surgery the monkey had been trained to perform a torque-tracking task (Maier et al., 1998). The monkey controlled the one-dimensional position of a cursor on a video monitor with isometric flexion and extension wrist torques, and acquired targets displayed on the screen. The monkey was required to maintain torque within each target for 0.5–1.0 s to receive a juice reward. Targets remained on the screen until the hold criterion was met, followed by presentation of the next target, either immediately or after a variable reward period.

### INTRACORTICAL MICROSTIMULATION

A few days after the cortical implant, movements evoked by intracortical microstimulation (ICMS) through the implanted electrodes with the monkey awake were documented by visual observation. Trains of 10 pulses of constant-current, biphasic square-wave pulses with 0.2-ms durations at 300 Hz evoked movements at thresholds of 20–120 μA (**Figure 1B**). The ICMS map was re-established 14 days after the spinal lesion with the monkey awake. After the spinal cord injury trains of ICMS at 450 μA were ineffective at most sites (**Figure 1C**).

### SPINAL STIMULATION PROCEDURE

Intraspinal stimuli consisting of constant-current, biphasic square-wave pulses with 0.2-ms durations were delivered through the spinal microwires. In general, stimulation (10–700 μA) was delivered by a single electrode. The output effects evoked from each spinal site were documented with stimulus-triggered averages of rectified EMG (St-TA) during task performance. Current pulses were delivered at a low rate (10–20 Hz) to avoid temporal summation. Stimulus-evoked facilitation and suppression of EMG were identified as consistent features in the St-TAs above or below, respectively, 2 standard deviations (SD) of baseline. Baseline was defined as the interval from 30 to 0 ms preceding the trigger pulse. The mean percent increase (MPI) measured the average values between onset and offset of the feature minus baseline, divided by baseline. Based on post-stimulus effects in St-TAs, we chose a single electrode and current for the artificial neural connection paradigm.

### BEHAVIORAL TASK WITH ARTIFICIAL NEURAL CONNECTION

Prior to establishing an artificial neural connection, the monkey learned to control a computer cursor with brain activity or muscle activity in separate operant conditioning sessions. Rack-mounted instrumentation was programmed to compile a running average (200 ms) of either EMG or rectified, high-gamma (90–160 Hz) LFP activity to create a continuous signal that controlled the one-dimensional position of a cursor on a video monitor. Targets that indicated high- or low-amplitude LFP or EMG were randomly presented on the screen. Targets remained on the screen until the monkey held the cursor within each target for 0.5–1.0 s to receive a juice reward.

After a few sessions of this preliminary task, an artificial connection to the spinal cord was established in subsequent sessions. Instead of directly controlling cursor position, LFP or EMG activity triggered spinal stimuli. Cursor position was now driven by isometric torque produced about the wrist. For each session, either flexion or extension torque controlled cursor position, depending on the torque produced by the spinal stimulation used in that session. The monkey learned to control LFP or EMG activity to acquire targets displayed on the monitor to receive a juice reward as described above (Maier et al., 1998). The direction of cursor movement was matched in all sessions; i.e., increases in LFP or EMG activity in preliminary sessions moved the cursor in the same direction as increases in torque during the sessions with an artificial connection.

### ARTIFICIAL CORTICOSPINAL CONNECTION

In several preliminary sessions, the monkey controlled the cursor with high-frequency gamma (90–160 Hz) LFP activity recorded in either M1 or PMd. Then an artificial corticospinal connection (ACSC) was established using the same signal which had been used in the previous preliminary sessions to trigger trains of spinal stimulation to bridge the impaired corticospinal connection. Rack-mounted instrumentation was programmed to compile a running average of rectified LFP activity in the high-gamma band and to trigger delivery of intraspinal microstimulation at 300 Hz to a single electrode whenever the LFP exceeded a threshold determined by the experimenter. Prior to each session, we determined the background noise level of the high-gamma band signal and set the threshold so that no stimulation was delivered when the monkey was at rest. St-TAs of EMG guided the choice of a single electrode in the spinal cord, the stimulus current, and the position of the cursor on the screen for the artificial neural connection. A few sessions of ACSC were tested within a day, but different pairs of cortical and spinal sites were chosen.

### ARTIFICIAL MUSCULOSPINAL CONNECTION

In several preliminary sessions, the monkey controlled the cursor with EMG activity recorded from a single forearm muscle. Even after the spinal cord lesion, the monkey could produce some muscle activity in the paretic hand. Then, an artificial musculospinal connection (AMSC) was established using the same EMG to trigger trains of spinal stimulation. Rack-mounted instrumentation was programmed to compile a running average of muscle activity and trigger a train of intraspinal microstimulation at 300 Hz during the time that EMG exceeded a threshold determined by the experimenter. Prior to each session, we determined the background noise level of the EMG signal and set the threshold so that no stimulation was delivered when the monkey was at rest. The stimulus-evoked EMG was insufficient to cross threshold, so the monkey could terminate stimulation by terminating his volitional EMG activity. Sessions of AMSC were tested on different days than ACSC. A few sessions of AMSC were tested within a day, but different pairs of muscles and spinal sites were chosen.

### HISTOLOGICAL PROCEDURES

At the end of the experiments, the monkey was deeply anesthetized with an overdose of sodium pentobarbital (50–75 mg/kg, i.v.) and perfused transcardially with 0.1 M phosphate-buffered saline (PBS, pH 7.3), followed by 4% paraformaldehyde in 0.1 M PBS (pH 7.3). The spinal cord was removed immediately and saturated with fresh PBS containing, successively, 10, 20, and 30% sucrose. Serial sections 50 μm thick were cut on a freezing microtome. Sections processed for Nissl staining with 1% cresyl violet were used to assess the extent of the lesion and the location of electrode tracks.

### STATISTICAL ANALYSIS

To determine the statistical difference of task performance between “artificial neural connection” and “catch” trials, we used the unpaired-*T* test. One-way analysis of variance (ANOVA) with repeated measures was performed to determine the significant differences in task performance among the M1, PMd, and M1 and PMd. *Post hoc* multiple comparisons were conducted using the Bonferroni test. Statistical significance level was set at *p* < 0.05. All pooled values are reported as mean ± SD.

## RESULTS

### EXTENT OF SPINAL LESION AND HISTOLOGIC EVIDENCE OF ELECTRODE LOCATION IN SPINAL CORD

**Figure 1D** shows the spinal cord section showing the maximum extent of the lesion located at the C2 level, as evidenced by gliosis. The dorsolateral region on the side ipsilateral to the lesion was severely deformed because of mechanical damage and degeneration of axons. The lesion area covered most of the right dorsolateral funiculus. The dorsal funiculi were partially damaged on both sides, but the ventrolateral funiculi were preserved on both sides. The lesion extended from the caudal part of C1 to most of C2. Thus, the lesion interrupted most of the corticospinal and rubrospinal tracts but preserved the reticulospinal tract and some ascending tracts.

We intended to position the electrode tips in the ventral horn and intermediate zone where motoneurons and premotoneuronal interneurons are located. We found two electrode tracks in the sections. **Figure 1E** shows one electrode track at the level of C6. The electrode tip was located in the ventral horn, as shown in **Figures 1Ea, b**. The second recovered electrode was located in the medial intermediate zone.

### FUNCTIONAL DEFICIT

The monkey’s ability to independently control movement of digits, such as for precision grip, exhibited deficits shortly after the lesion and did not recover throughout the 3-month experimental period. Power grip recovered gradually 5–7 weeks after the lesion, consistent with a previous study (Alstermark et al., 2011).

The cortical somatotopic maps before and after lesion are shown in **Figures 1B, C**. Movements could be evoked from only two of five previously effective sites in M1, and only with higher currents than before the injury. The PMd was even more affected since no movements at all could be elicited from two previously effective sites after the lesion. Thus the extent and excitability of the upper limb representation in motor cortex decreased substantially after the spinal lesion, consistent with a previous study (Schmidlin et al., 2004).

### SPINAL STIMULATION

**FIGURE 2:**
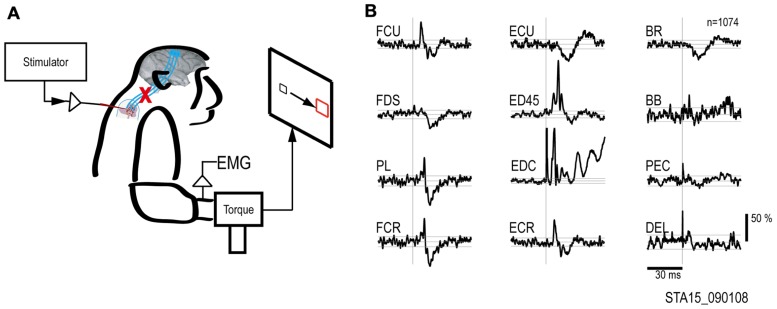
**Output effects evoked by intraspinal stimulation.** **(A)** Electrical stimuli were delivered to a single intraspinal electrode while the monkey performed a two-dimensional wrist task, acquiring targets in wrist flexion and extension. **(B)** Muscle responses evoked by a single pulse at 90 μA. The vertical scale bar at right indicates mean percent increase (MPI) over baseline. EMGs were recorded from: flexor carpi ulnaris (FCU), flexor digitorum superficialis (FDS), palmaris longus (PL), flexor carpi radialis (FCR), extensor carpi ulnaris (ECU), extensor digitorum 4 and 5 (ED45), extensor digitorum communis (EDC), extensor carpi radialis (ECR), brachioradialis (BR), biceps brachii (BB), pectoralis (PEC), and deltoid (DEL).

To document the muscle responses evoked by intraspinal stimuli we compiled St-TAs of rectified EMG during performance of the wrist flexion and extension task. **Figure 2** shows the St-TAs for a spinal site located caudal to the lesion. Spinal stimulation below the lesion evoked facilitation or suppression effects in multiple muscles, as found for effects evoked from the intact spinal cord (Moritz et al., 2007). Furthermore, spinal stimulation activated synergistic muscle groups. For example, stimuli at the site in **Figure 2** strongly facilitated finger extensor muscles [e.g., extensor digitorum 4 and 5 (ED45) and extensor digitorum communis (EDC)] and suppressed antagonist flexor muscles. Facilitation or suppression effects were evoked in 45.6% of the 12–16 recorded muscles from all spinal sites. Based on the responses in St-TAs, we chose a single electrode and current for the artificial neural connection paradigm. For all electrodes, stimulus effects gradually deteriorated over 3 months, presumably due to electrode encapsulation by the physiological reaction (Mushahwar et al., 2000). Finally, the whole spinal implant including wire electrodes with dental acrylic and bone screws sloughed off the vertebrae after 3 months.

### ARTIFICIAL CORTICOSPINAL CONNECTION

**FIGURE 3:**
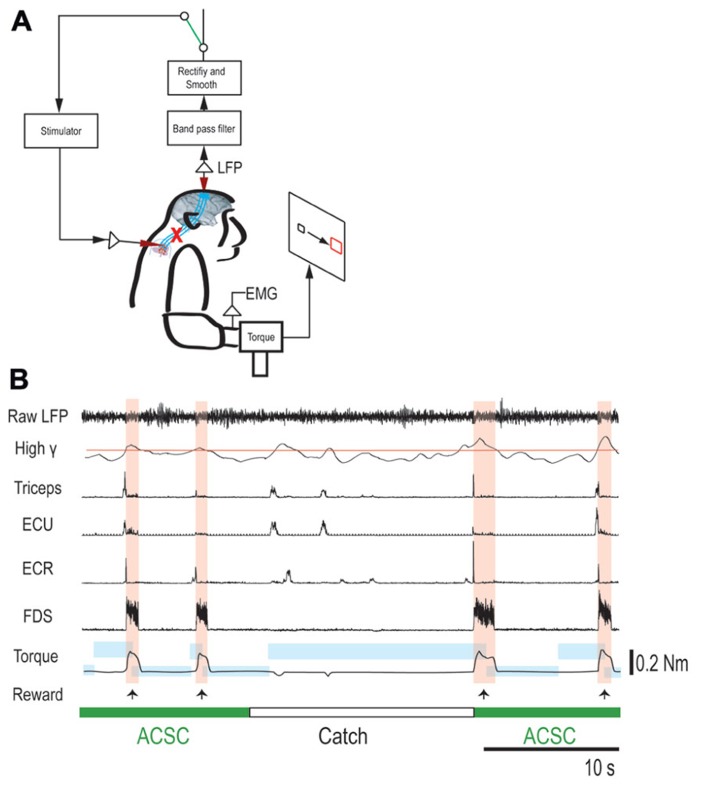
**Brain-controlled intraspinal stimulation below the lesion.** **(A)** Schematic shows local field potential (LFP) in motor cortex gating trains of electrical stimulation (300 Hz) to a spinal site below the lesion. The switch in the recurrent loop was opened for catch trials. **(B)** Four successful trials with the artificial corticospinal connection (ACSC, green) and one catch trial (white). During the catch trial, the monkey made several unsuccessful attempts to produce wrist torque, as seen in the EMG and torque. The blue rectangles indicate duration and force range of target. The pink vertical bars indicate duration of electrical stimulation in the spinal site. The red line in second trace represents the threshold for spinal stimulation. From top, raw LFP in motor cortex, rectified and smoothed high-gamma LFP (90–160 Hz), EMG from four muscles (abbreviations as in **Figure 2**), and wrist torque. Arrows indicate times of successful task completion and reward.

**FIGURE 4:**
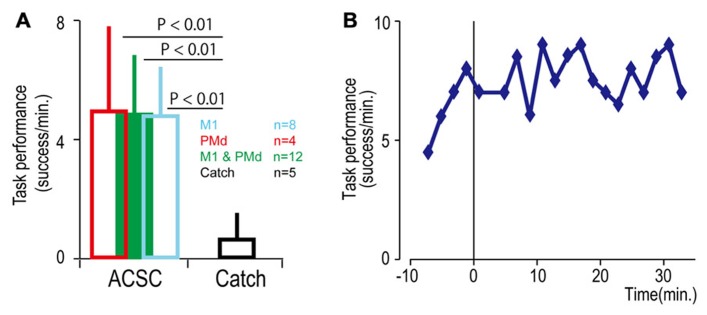
**Task performance in the artificial corticospinal connection (ACSC).** **(A)** Average task performance with the ACSC and during catch trials. Error bars represent standard deviation. **(B)** Time course of task performance. Before time zero the monkey was required to control the cursor with LFP activity. After time zero the task involved ACSC, using the same cortical electrode in digit area of M1.

To bridge the spinal cord lesion, high-gamma LFP activity in either M1 or PMd was used to trigger trains of spinal stimulation (**Figure 3A**). **Figure 3B** shows a typical example of intraspinal stimulation controlled by the LFP signal recorded from the digit area of M1 (site identified by arrow in **Figure 1A**). During the period of FES (green bar), the monkey was able to trigger and stop stimulation volitionally, thereby repeatedly acquiring the targets. To document that the LFP-controlled intraspinal stimulation was necessary, the stimulation was briefly turned off during “catch trials” (white bar in **Figure 3B**) in five sessions. The monkey continued to make efforts to acquire the target in the catch trials, as evidenced by the above-chance increases in high-gamma activity, but did not succeed in acquiring the targets. We applied such LFP-controlled intraspinal stimulation in 12 different sessions (duration of sessions: 8–47 min; range of trial number within each session: 46–245 trials), using 11 different pairs of cortical and spinal sites, summarized in **Figure 4A**. The average task performance in LFP-controlled intraspinal stimulation trials was significantly higher than those in catch trials (compare green and black bars in **Figure 4A**). Task performance was comparable with LFP recorded from M1 and PMd (cf. blue and red bars in **Figure 4A**). Task performance using LFP from cortical sites from which movements could and could not be evoked after injury was similar. We also examined the task performance during the transition from the operant conditioning of LFP to ACSC. **Figure 4B** shows the time course of task performance in the operant conditioning session (before time zero in **Figure 4B**) and subsequent ACSC session (after time zero in **Figure 4B**) using LFP from the same cortical electrode. The monkey quickly learned after a few sessions to modulate the power of LFP to acquire targets. Switching from the operant control sessions to the ACSC session was very smooth. The task performance in the ACSC session was sustained at nearly the same level as in the operant conditioning session.

### VOLITIONAL BOOSTING OF MUSCLE ACTIVITY BY AN ARTIFICIAL MUSCULOSPINAL CONNECTION

**FIGURE 5:**
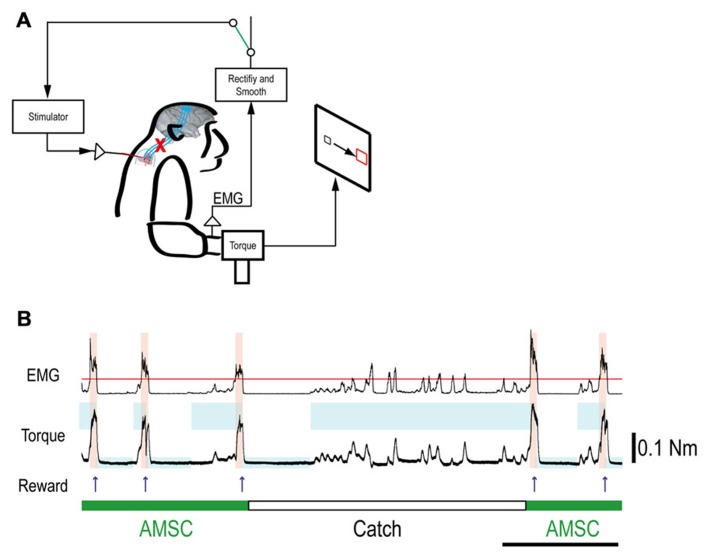
**Muscle-controlled spinal cord stimulation.** **(A)** Schematic shows EMG activity gating a train of stimuli to a spinal site below the lesion. **(B)** Five successful trials with AMSC (green) and unsuccessful catch trials (white). During the catch trial, the monkey made several unsuccessful attempts to produce wrist torque, as seen in the EMG and torque. The blue rectangles indicate duration and force range of target. The pink bars indicate duration of electrical stimulation in the spinal site. The red line in top row represents the threshold for gating spinal stimulation. The upper and lower traces are the EMG from ECR and wrist torque generated by the monkey, during stimulation (AMSC, in green) or without stimulation (Catch, in white). Arrows indicate times of successful task completion and reward.

**FIGURE 6:**
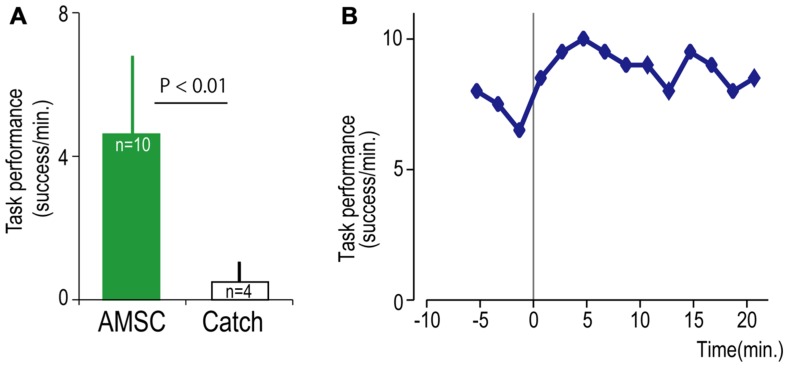
**Task performance with the artificial musculospinal connection (AMSC).** **(A)** Average task performance for AMSC and catch trials. Error bars represent standard deviation. **(B)** Time course of task performance in AMSC session. Before time zero the monkey controlled the cursor with EMG activity. After time zero the monkey controlled wrist torque via spinal stimulation triggered from the same muscle with the AMSC.

Although the spinal cord lesion produced a severe deficit in forearm movements, the monkey could still produce weak muscle activity. To investigate whether an artificial recurrent connection could boost the activity of a muscle, we used EMG of the paretic muscles to trigger spinal stimulation at a site that produced a contraction of the same muscle (**Figure 5A**). **Figure 5B** shows a typical example of the muscle-controlled intraspinal stimulation using EMG of the paretic extensor carpi ulnaris (ECU) wrist extensor muscle. As shown during the period of FES (green bar), the monkey was able to sustain the EMG burst and torque to acquire the target. During the catch trial, the monkey made a few unsuccessful attempts to produce wrist torque, as seen in the EMG and torque, but was unable to acquire the target. Thus, the muscle-controlled intraspinal stimulation effectively boosted on-going muscle activity of the paretic agonist. We applied similar muscle-controlled FES in 10 different sessions (duration of sessions: 12–33 min; range of trial number within each session: 42–180 trials), using five different pairs of muscle and spinal sites. The average task performance in muscle-controlled intraspinal stimulation trials was significantly higher than that in catch trials (compare green and black bars in **Figure 6A**). **Figure 6B** shows the time course of task performance in the session of operant conditioning of EMG activity and subsequent AMSC session using EMG from the same muscle. The task performance in AMSC sessions was sustained at nearly same level as with operant conditioning session.

## DISCUSSION

This case report demonstrates that LFP- or EMG-controlled stimulation in a spinal site could be used to produce volitionally controlled functional wrist torque in a paretic monkey with a spinal cord lesion rostral to the stimulation site. The monkey could volitionally control brain and muscle activities to produce synergistic muscle responses with intraspinal stimulation caudal to the lesion. These results suggest that muscle- or LFP-controlled FES could compensate for the interrupted descending pathways and restore volitional control of functional movement in the upper limb after spinal cord injury or stroke.

The fact that stimulation in a spinal site caudal to a spinal cord lesion can evoke synergistic muscle responses suggests that activity-dependent spinal stimulation may be a promising target for neuroprosthetics that can restore movements after spinal cord injury. In contrast to FES of muscles, spinal microwires are subject to less mechanical fatigue than wires implanted peripherally and require lower stimulus currents to evoke movements. Intraspinal stimulation also produces more natural, graded recruitment of motor units than muscle or nerve stimulation (Mushahwar and Horch, 1998). We found that spinal stimulation caudal to the lesion evoked facilitation or suppression effects in multiple muscles. Intraspinal stimulation is known to activate many afferent fibers of passage (Gaunt et al., 2006), and probably excites motoneurons transsynaptically by activating a sufficient number of their inputs, such as propriospinal, corticospinal, and/or afferent axons. Fibers of passage have lower activation thresholds than cell bodies and are thus recruited at lower stimulus currents (Gustafsson and Jankowska, 1976). Afferent axons directly excite synergist muscles and inhibit antagonist muscles via inhibitory spinal interneurons. Since we used a single signal, derived from either cerebral cortex or muscle, to control stimulation, the degree of movement control demonstrated here remains limited. Extending this strategy to control more natural and complex movements would require additional input signals and output spinal sites. Compared with FES in muscle, the activation of functional muscle synergies from single intraspinal sites could significantly reduce the number of implanted electrodes as well as the number of independent control signals required from a neuroprosthetic system.

Task performance with LFP recorded in M1 was comparable with performance using LFP from PMd or EMG from muscle. Furthermore, LFP from any cortical site could control spinal stimulation-evoked wrist movements, regardless of whether stimulation of the cortical site evoked wrist movements or not (cf. **Figure 1C**). Previous biofeedback studies have shown that cells in motor (Fetz and Baker, 1973; Fetz and Finocchio, 1975; Moritz et al., 2008) or somatosensory (Moritz and Fetz, 2011) cortex with no discernable relation to muscles can be volitionally modulated after brief practice sessions. We used a similar operant conditioning paradigm with biofeedback for eliciting cortical LFP or EMG to trigger spinal stimuli. The level of performance in the operant conditioning task was identical to that with the ACSC and AMSC artificial neural connections. Thus, an arbitrary cortical or muscle signal could be brought under volitional control using biofeedback, to substantially expand the sources of control signals for brain–computer interfaces.

Implementation of the artificial connections with a portable bidirectional neural interface will enable adaptive learning over much longer times and under more varied conditions (Jackson et al., 2006b; Nishimura et al., 2010). The autonomous ‘Neurochip’ system can discriminate brain or muscle activity and deliver stimulation during free behavior (Zanos et al., 2011). Such autonomous low-power circuits could allow subjects to practice continuously with an artificial connection, without requiring complex decoding algorithms or external devices such as robotic arms. Further development of such direct control of a paretic extremity may lead to implantable devices that could help restore volitional movements to individuals with impaired motor control. Furthermore, recent evidence suggests that continuous activity-dependent stimulation promotes plasticity in motor cortex (Jackson et al., 2006a) and corticospinal connections (Fetz et al., 2010). Thus, activity-dependent stimulation during free behavior may produce both adaptive learning to exploit artificial connections (Nishimura et al., 2010) as well as Hebbian strengthening of spared pathways after neural damage in descending pathway (Fetz et al., 2010). Furthermore, long-term exposure to artificial neural connections could induce reorganization of cortical and spinal circuitry and facilitate functional recovery.

In conclusion, this study demonstrates that artificial neural connections that bridge impaired pathways can ameliorate functional loss. Closed-loop control with intraspinal microstimulation driven by brain or muscle activity could control synergistic muscle activities in upper limb in a monkey with spinal cord injury. The success of our protocol suggests that neurorehabilitative treatment could exploit similar paradigms for restoring volitional control of the extremity for individuals with spinal cord injury or stroke.

## CONTRIBUTIONS

Yukio Nishimura and Steve I. Perlmutter conducted the experiments. Yukio Nishimura analyzed the data. Yukio Nishimura, Steve I. Perlmutter, and Eberhard E. Fetz conceived the study and wrote the manuscript.

## Conflict of Interest Statement

The authors declare that the research was conducted in the absence of any commercial or financial relationships that could be construed as a potential conflict of interest.
